# Longitudinal white matter alterations in SIVmac239-infected rhesus monkeys with and without regular cART treatment

**DOI:** 10.3389/fimmu.2022.1067795

**Published:** 2023-01-12

**Authors:** Jiaojiao Liu, Benedictor Alexander Nguchu, Dan Liu, Yu Qi, Xire Aili, Shuai Han, Yuxun Gao, Xiaoxiao Wang, Hongwei Qiao, Chao Cai, Xiaojie Huang, Hongjun Li

**Affiliations:** ^1^ Department of Radiology, Beijing YouAn Hospital, Capital Medical University, Beijing, China; ^2^ Center for Biomedical Imaging, University of Science and Technology of China, Hefei, China; ^3^ Department of Radiology, Renji Hospital, Shanghai Jiaotong University School of Medicine, Shanghai, China; ^4^ Beijing Advanced Innovation Centre for Biomedical Engineering, Beihang University, Beijing, China; ^5^ Institute of Laboratory Animal Sciences, Chinese Academy of Medical Sciences, Beijing, China; ^6^ Beijing YouAn Hospital, Capital Medical University, Beijing, China; ^7^ Clinical and Research Center for Infectious Diseases, Beijing YouAn Hospital, Capital Medical University, Beijing, China

**Keywords:** SIV-mac239 infected Chinese rhesus monkeys, diffusion tensor imaging, white matter changes, combined antiretroviral therapy, CD4/CD8 ratio, viral load

## Abstract

**Objective:**

To use SIV-mac239-infected Chinese rhesus monkeys to study white matter changes with and without regular combined antiretroviral therapy (cART) and the relationships between the changes and clinical results.

**Methods:**

Diffusion tensor imaging (DTI) data were collected at baseline and 10 days, 4 weeks, 12 weeks, 24 weeks, and 36 weeks after viral inoculation. Plasma CD4 T cell counts, CD4/CD8 ratio, plasma viral load, and cerebrospinal fluid (CSF) viral load were collected at baseline and 1 week, 5 weeks, 12 weeks, 24 weeks, and 36 weeks after viral inoculation. Microstructural characteristics were examined within 76 white matter areas defined by the DTI-white matter (WM) atlas for rhesus macaques. Corrections for multiple comparisons were performed using a false discovery rate (p < 0.05, FDR). Correlation analyzes between imaging markers and clinical markers (plasma CD4 T cell counts, CD4/CD8 ratio, plasma viral load, and cerebral spinal fluid viral load) were performed using Pearson correlations.

**Results:**

White matter changes in SIV-infected macaques were detected in different brain regions as early as 4 weeks after inoculation. As time progressed, cART reversed, ameliorated, or even enhanced the effects. The CD4 T cell count was mainly associated with DTI metrics before cART, while the CD4/CD8 ratio was associated with white matter changes with and without cART. Viral load was positively associated with mean diffusivity in HIV patients without cART, and the opposite results were seen in HIV patients with cART.

**Conclusion:**

SIV-mac239 infection may be an ideal tool for studying HIV-induced changes in the brain. The first white matter changes appeared in a structure adjacent to the periventricular area as early as 4 weeks after inoculation. As time progressed, cART had different effects on different regions, reversing, attenuating, or even progressing the pathology. Moreover, these changes were closely related to the CD4/CD8 ratio and viral load, even after cART.

## 1 Introduction

HIV can enter the central nervous system (CNS) shortly after seroconversion and activate microglial and macrophage cells to release toxic factors such as excitotoxins, viral products, cytokines, and chemokines. These lead to marked inflammation, immune activation and suppression, and disruption of the blood-brain barrier (1–3) and result in neuronal damage that is visible as changes in brain white matter structure. The use of combination antiretroviral therapy (cART) has transformed HIV from a fatal disease into a chronic, manageable disease (4) and can reverse and prevent milder impairments (ANI and MND) to some extent. However, also of concern are the iatrogenic effects of the drugs on brain integrity (5, 6). For example, previous studies have demonstrated axonal dysfunction and synaptic damage in HIV patients (7, 8). Diffusion tensor imaging (DTI) is a widely used noninvasive neuroimaging technique that is very sensitive to microstructural changes (9). Previous studies have shown decreased fractional anisotropy (FA) and increased mean diffusivity (MD) throughout the brain of HIV-infected patients (10–21), even those on effective antiretroviral therapies (7, 11). However, studies have also found no significant differences between HIV-infected patients and healthy controls (22), and some investigators noted that the use of cART does not appear to prevent or reverse existing brain damage (23–25). However, most of these studies were cross-sectional and involved multiple factors, including route of infection, inclusion criteria, duration of infection, complications, different treatment regimens, and lack of patient adherence, which are difficult to control for (13, 17, 18). Because most changes occur months or even years after infection, studies to investigate early changes in the brain of HIV-infected individuals are needed. Therefore, longitudinal studies with controlled covariances should be conducted to address the above problem and shed light on the mechanisms underlying the dynamic changes that are seen with and without the use of cART.

An appropriate animal model whose pathological mechanisms are similar to those of HIV patients can be used to clarify this issue. Studies have shown that the pathology of simian immunodeficiency virus (SIV)-infected Chinese rhesus monkeys is very similar to that of HIV-infected patients, especially in terms of central nervous system (CNS) dysfunction, cognitive or behavioral deficits, and the development of AIDS (26–28). Moreover, previous DTI studies have shown SIV-infected rhesus macaques to have metric abnormalities similar to those seen in HIV patients (29, 30).

In the present study, we dynamically investigated white matter changes in SIV-mac239-infected Chinese rhesus monkeys with and without regular cART treatment and the relationship between DTI metrics and clinical tests (including plasma CD4 T cell counts, CD4/CD8 ratio, plasma viral load, and CSF viral load) at different time points.

## 2 Materials and methods

### 2.1 Animal experiments before the main experiments

This study was approved by the Beijing Municipal Sciences & Technology Commission. Ten healthy male Chinese-origin rhesus monkeys (age, 3.8 ± 0.3 years; weight, 4.7 ± 0.6 kg) were applied in the study. Before conducting the longitudinal study, health screening and indirect immunoassay (IFA) were performed on all rhesus monkeys to confirm their healthy status and exclude possible infection with simian immunodeficiency virus (SIV), simian type D retrovirus (SRV), herpes B virus, or simian T cell lymphotropic virus-I (STLV-I).

### 2.2 Animal model of SIV-infected rhesus monkey and data collection

**Figure 1:**
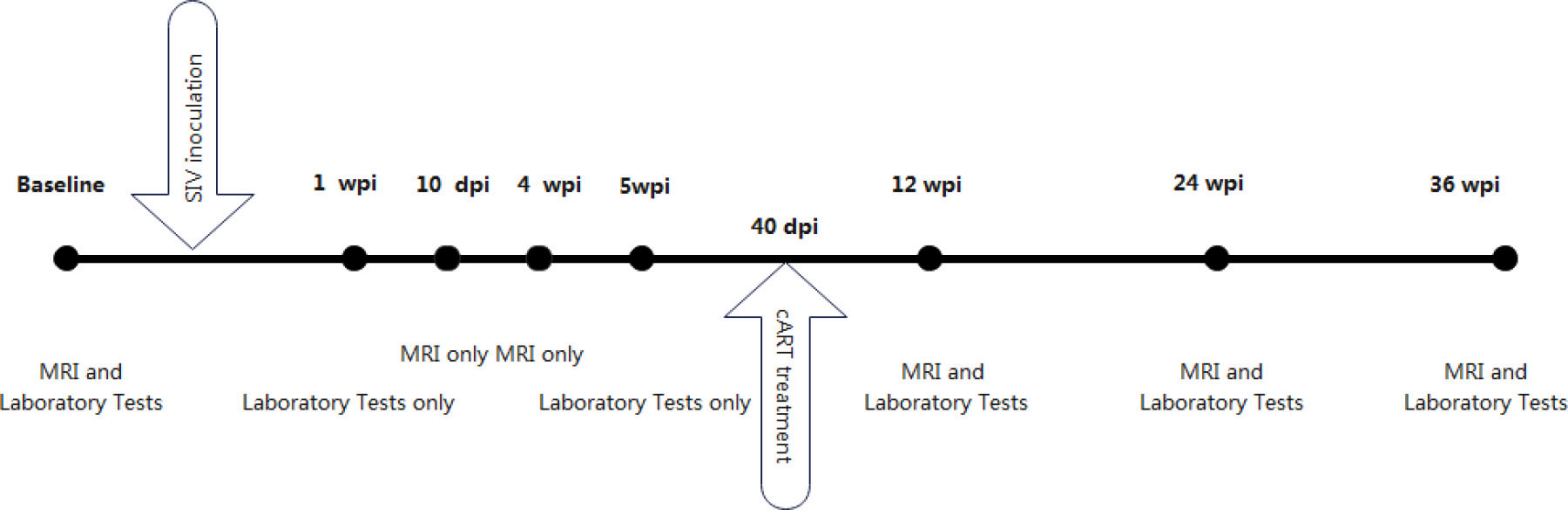
Timeline of macaques analysis in our study.

Ten macaques were inoculated intravenously with a 50-fold half-tissue culture infective dose (TCID50) of SIVmac239 through the brachial vein and were all seroconverted to SIV within 7 days of inoculation. Five of them received cART 40 days after inoculation, while the other five macaques received no treatment. The treatment regimen included emtricitabine (50 mg/kg per day), tenofovir disoproxil fumarate (5.1 mg/kg per day), and dolutegravir (2.5 mg/kg per day). Baseline was determined 2 weeks before inoculation. Immunologic characteristics of peripheral blood, including plasma CD4 T cell counts, CD4/CD8 ratio, plasma viral load, and CSF viral load, were assessed at baseline and 1 week, 5 weeks, 12 weeks, 24 weeks, and 36 weeks after SIV-mac239 inoculation. The CD4/CD8 ratio was calculated based on T cell counts. MRI scans were performed at baseline and 10 days, 4 weeks, 12 weeks, 24 weeks, and 36 weeks after virus inoculation (**Figure 1**).

### 2.3 Housing conditions for experimental subjects

All animals were housed at the Institute of Laboratory Animal Sciences, Peking Union Medical College, under the same housing conditions as in previous studies (7, 21), i.e., temperature of 16–26°C, humidity of 40%–70%, 12h/12h light/dark cycle, water ad libitum, and twice-daily feeding without dietary restrictions.

### 2.4 DTI data analysis

#### 2.4.1 MR Protocol

MRI scans were performed at Beijing YouAn Hospital, Capital Medical College, using a 3T Siemens Tim TRIO whole-body magnetic resonance imaging scanner (Siemens, Germany) at each time point to assess the longitudinal effects of SIV infection on the brain. The monkeys were anesthetized and were in the supination position during the MRI scans. DTI images were acquired with gradient-echo single-shot echo planar imaging (EPI). The parameters were repeat time/echo time (TR/TE) = 5200/100 ms, FOV = 152 mm × 152 mm, data matrix = 76 × 76, flip angle = 90°, slice thickness = 2 mm (voxel size = 2 × 2 × 2 mm^3^), and time points = 10 min 52 s.

#### 2.4.2 Macaque image processing

**Figure 2:**
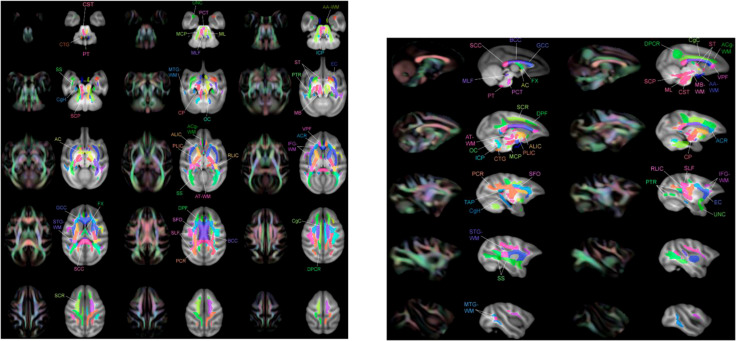
Multiple layers of the atlas overlaid with T1-W template showing regions of interest (ROIs) adjacent to the color image FA. These ROI images were taken from Zakszewski et al. (32).

Preprocessing of macaque DWI data was performed in accordance with previous studies using FMRIB Software Library (FSL) (https://fsl.fmrib.ox.ac.uk/) and AFNI (3D Skull Strip for skull stripping of monkey brain data). The raw DWI data were preprocessed to correct for eddy currents, susceptibility-related biases, and animal motion. The data were then fitted using the DIT-tensor fitting technique available in FSL. Four types of diffusivity maps were created: fractional anisotropy (FA), a measure of the directionality of water diffusion; mean diffusivity (MD), a measure of water diffusivity in the transverse directions (31). Each of these macaque diffusivity maps (FA, MD) was applied to the standard spatial template, the diffusion tensor-based white matter atlas for rhesus macaques (also known as UWDTIRhesusWMAtlas, https://www.nitrc.org/projects/rmdtitemplate/) (32) (**Figure 2**) using FMRIB Linear/Non-Linear Image Registration Tools (FLIRT/FNIRT), part of FSL version 5.09 (33). The template is population-based and was developed from a large number of high-quality scans of animals (N = 271), allowing it to account for variability in animal species. It has a high signal-to-noise ratio (SNR) and FA values, as well as high image sharpness with visible small white matter structures and spatial features (32). Two neurologists (X.W. and B.N., with 8 and 4 years of experience, respectively) visually reviewed the data to confirm the accuracy of the registration.

#### 2.4.3 Selection of areas of interest (ROIs) and WM analysis of microstructural features

Microstructural properties were examined within the 76 white matter areas defined by the DTI-WM atlas for rhesus monkeys. All WM structures were assessed in our analysis. Each WM region of interest (ROI) was examined longitudinally for changes over time. These microstructural changes were examined at baseline and at 10 days, 4 weeks, 12 weeks, 24 weeks, and 36 weeks after virus inoculation.

#### 2.4.4 Statistical analyses

Statistical analyses were performed using SPSS software [IBM SPSS Statistics for Windows, version 20.0 (IBM Corporation, Armonk, NY, USA)]. Group differences between macaques with cART and those without cART, the effect of time on microstructural characteristics, and the interaction effects of cART treatment and time were determined using a two-factor mixed design ANOVA. Of the 10 HIV-infected monkeys, five were randomly assigned to cART treatment, and the other five received no treatment. Thus, the between-subject factor had two levels. Over a 36-week period, microstructural characteristics were measured at six time points corresponding to six levels of the within-subject factor. These stages were baseline [time 1], after 10 days [time 2], after 4 weeks [time 3], after 12 weeks [time 4], after 24 weeks [time 5], and after 36 weeks at the end of the program [time 6].

Corrections for multiple comparisons were performed using a false discovery rate (p < 0.05, FDR). Correlation analyses between imaging markers and clinical measures (CD4 T cell count, CD8 T cell count, CD4/CD8 ratio, plasma viral load, and CSF viral load) were determined using Pearson correlations.

## 3 Results

### 3.1 Viral load, CD4 T cell count, and CD4/CD8 ratio trajectories in three groups

Differences in age and weight at baseline were not significant (age, P = 0.258; weight, P = 0.309). A significant time by group interaction was detected for weight (P < 0.001), plasma viral load (P < 0.001), CSF viral load (P < 0.001), peripheral blood CD4 T cell count (P < 0.001), and CD4/CD8 ratio (P < 0.001), which supported increasingly divergent trajectories among three groups.

A sharp increase in viral load was observed, and viremia arrived at its peak within 2 weeks in almost all SIV infected macaques. Plasma viral load in macaques without treatment subsequently declined and reached a plateau in 5 weeks. Macaques with cART treatment showed a dramatic increase within 5 weeks, after which they showed a continuous decline.

CSF viral load decreased to nadir at 24 weeks post-infection (wpi) and rose again afterward in the group without treatment. With cART, the viral load in the CSF dropped more dramatically and was undetectable in three macaques at 36 wpi.

There were reductions in CD4 T cell count and CD4/CD8 ratio with SIV infection that were alleviated by cART. After the introduction of cART, the CD4/CD8 ratio showed a tendency to increase.

**Figure 3:**
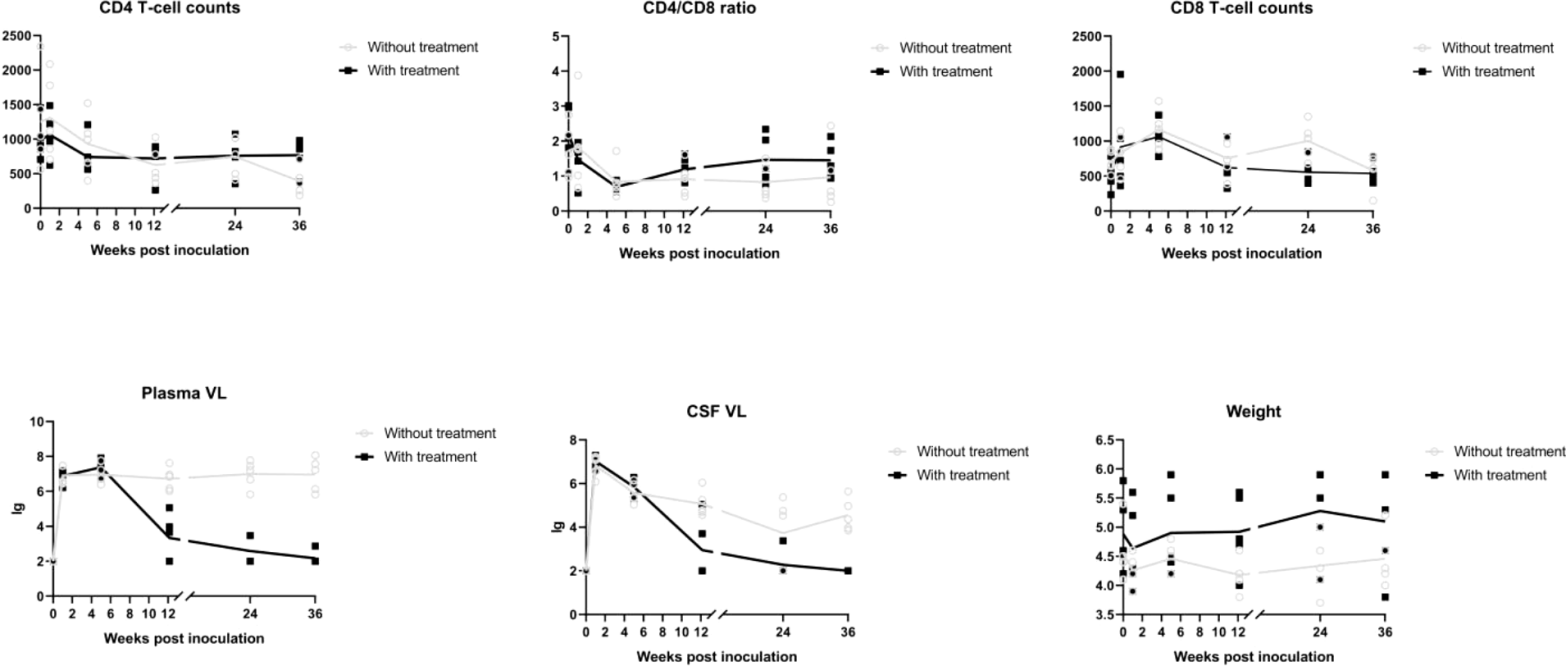
Alterations of CD4 T cell counts, CD8 cell counts, CD4/CD8 ratio, plasma viral load, CSF viral load in different time points of macaues with and without cART treatment.

Weight loss was seen in the SIV-cART group at 5 wpi, and the SIV-cART+ group showed a slight weight increase starting at 12 wpi (**Figures 3**).

### 3.2 Longitudinal DTI changes in macaques without treatment

**Figure 4:**
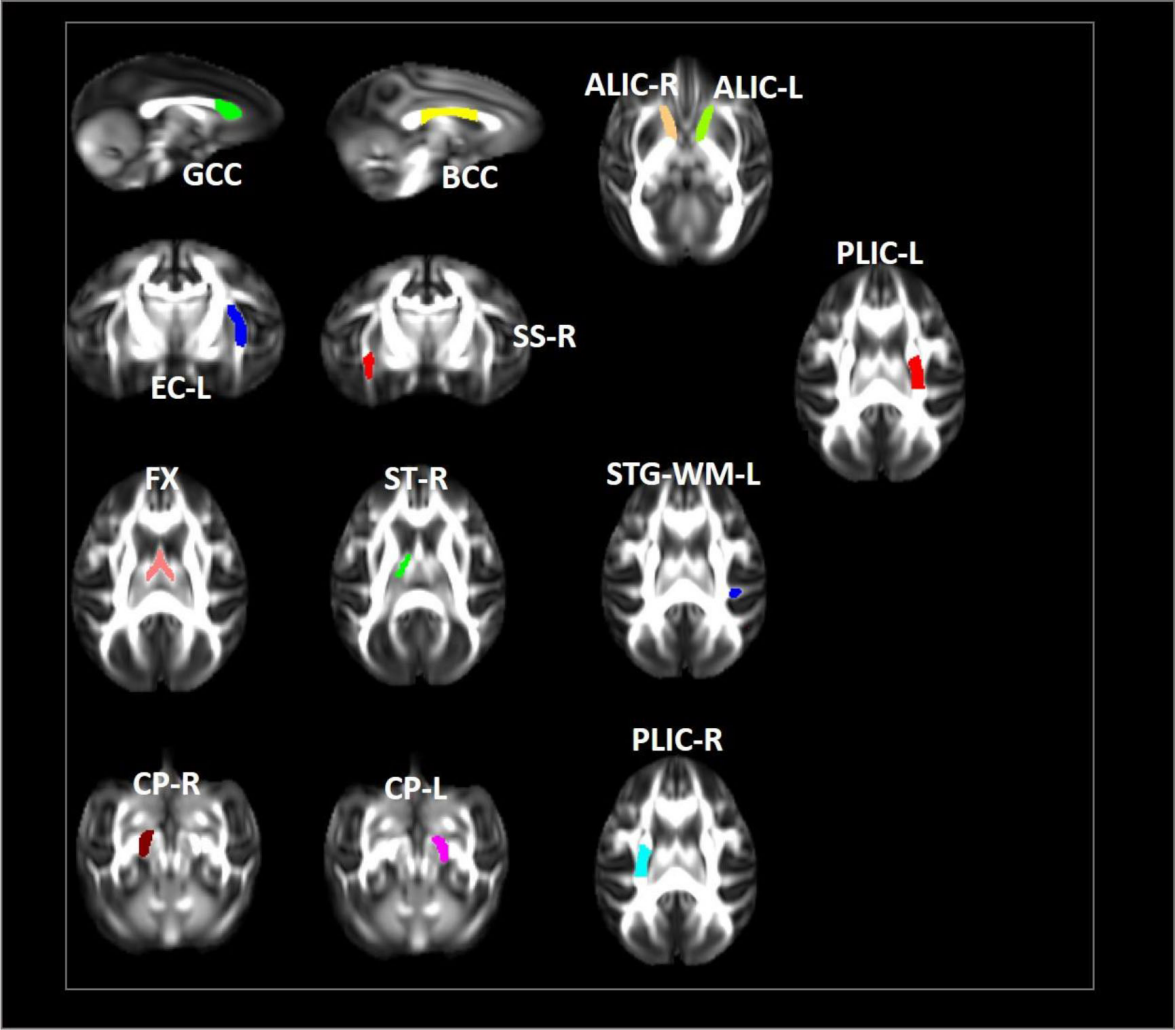
Changes in microstructures propagate differentially in SIV-macaques with and without antiretroviral therapy.

**Figure 5:**
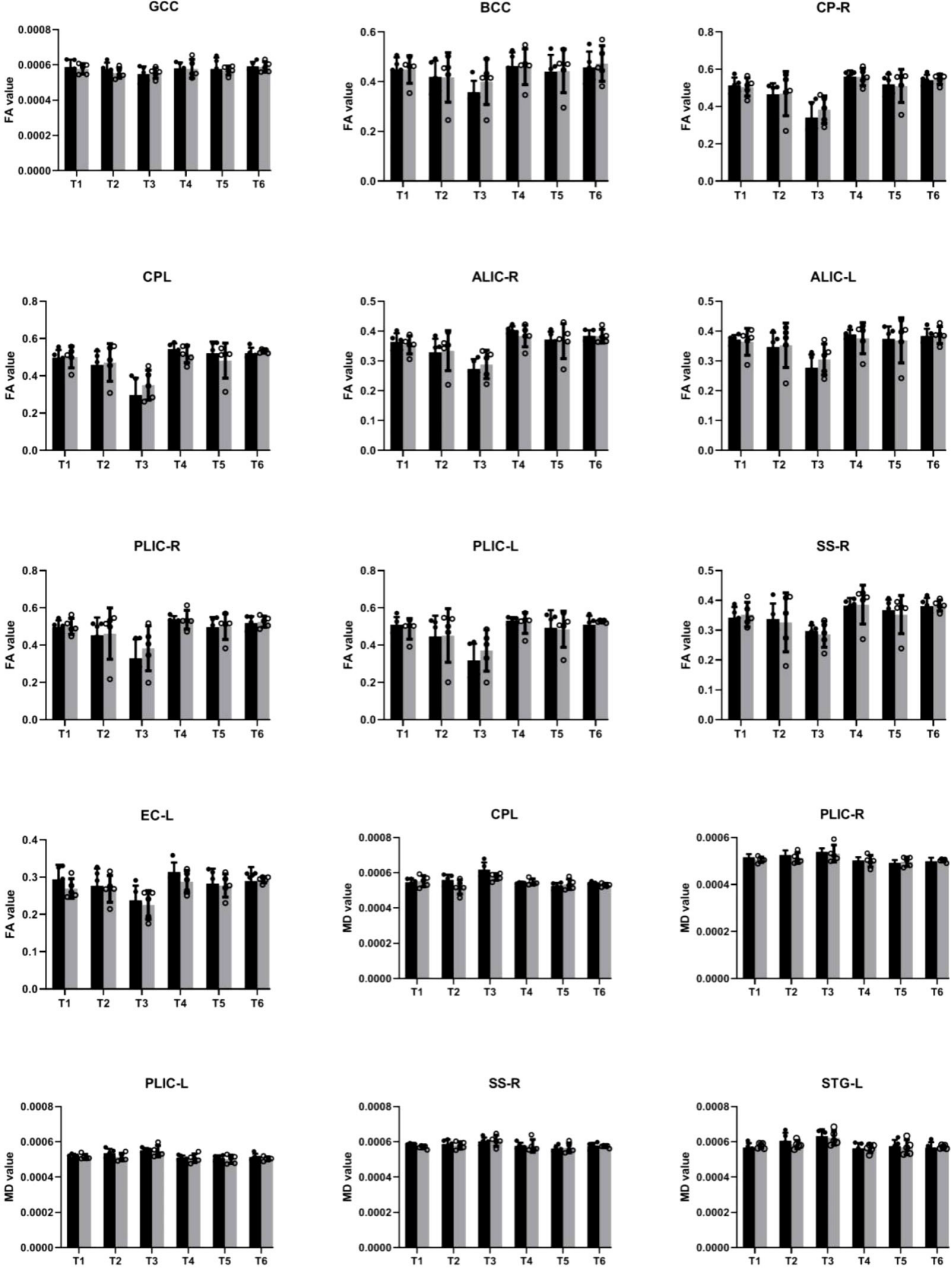
Illustration of significant alterations of FA and MD across time in cART- and cART+ groups. Bars, mean ± SE. Black, SIV-mac239 infected macaques without treatment; Gray, SIV-mac239 infected macaques with regular treatment. Note: T1 the baseline; T2, 10 days post virus inoculation; T3, the 4th week post virus inoculation; T4, the 12th week post virus inoculation; T5, the 24th week post virus inoculation; T6, the 36th week post virus inoculation. GCC, Genu of Corpus Callosum; BCC, Body of Corpus Callosum; CP-R, Cerebral Peduncle - Right; CP-L, Cerebral Peduncle - Left; ALIC-R, Anterior Limb of the Internal Capsule – Right; ALIC-L, Anterior Limb of the Internal Capsule – Left; PLIC-R, Posterior Limb of the Internal Capsule – Right; PLIC-L, Posterior Limb of the Internal Capsule – Left; SS-R, Sagittal Striatum - Right; EC-L, External Capsule - Left; STG-L, Superior Temporal Gyrus – Left.

In our study, we examined longitudinal white matter changes from baseline and 1 wpi, 4 wpi, 12 wpi, 24 wpi, and 36 wpi. We found that changes were first seen 4 wpi in the insuperior temporal gyrus (STG) (p = 0.029244), posterior part of the internal capsule (left) (PLIC-L) (p = 0.023222), fornix (FX) (p = 0.041225), left brainstem (p = 0.0173969), right brainstem (p = 0.024109), left anterior limb of the internal capsule (ALIC-L) (p = 0.0162715), right anterior limb of internal capsule (ALIC-R) (p = 0.01556), and right sagittal striatum (SS-R) (p = 0.0190229) at FA, and in the adjacent white matter of the amygdala (AA-WM-RR) (p = 0.004665), posterior limb of the internal capsule – left (PLIC-L) (p = 0.0232217) (p = 0.0451514), and right sagittal striatum (SS-R) (p = 0.0292208) on MD. By 24 weeks after inoculation, changes had occurred in the posterior limb of the right internal capsule (PLIC-R) (p = 0.0426243), corpus callosum (GCC) (p = 0. 0192415), body of the corpus callosum (BCC) (p = 0.032449), outer capsule – left (EC-L) (p = 0.01540547) (p = 0.0447482), and striaterminalus – right (ST-R) at FA (p = 0.0029465), and PLIC-R (p = 0.016532) (p = 0.0328549) at MD (**Figures 4**, **5**).

### 3.3 Long-term DTI changes in macaques under regular cART

After cART, we found no changes in BCC or FX for FA and MD; no progression in AA-WM R for MD or PLIC-R and PLIC-L for FA; slow progression in STG-WM-L, GCC, CP-R, and CP-L; and no effects on PLICR, ALIC-R, SS-R, or ST-R (**Figures 4**, **5**).

### 3.4 Correlation between altered DTI properties with regular cART and clinical measurements

We examined the relationships between clinical measurements, such as plasma CD4 T cell count, CD4/CD8 ratio, plasma viral load, and CSF viral load, and altered DTI metrics with high significance in macaques treated with cART. We confirmed that increased MD and FA were associated with decreased CD4T cell counts in only one region in AA-WM-R, and only a positive relationship was seen for ST-R. Additionally, MD was associated with a low CD4/CD8 ratio in GCC, ALIC-R, PLIC-L, and ALIC-L, and there were positive relationships with the ratio in ST-R and AA-WM-R, with regular cART treatment. CP-R and CP-L showed positive correlations with FA (**Table 1**). In addition, for the plasma viral load, MD was negatively correlated with brain regions in PLIC-L, PLIC-R, GCC, BCC, and SS-R. Finally for the CSF viral load, MD was positively related to CP-R, whereas there were negative relationships between FA and ALIC-R, ALIC-L, ST-R, GCC, BCC, PLIC-L, PLIC-R, FX, CP-R, CP-L, and SS-R (**Table 2**).

**Table 1:** The correlations between DTI metrics and clinical tests with regular cART treatment.

	Target region	CD4		CD4/CD8	
		r	p	r	p
MD	GCC	NA	NA	(T5)-0.8852612	0.045843
	ALIC-R	NA	NA	(T5)-0.86564256	0.057912
	PLIC-L	NA	NA	(T5)-0.94726	0.0144217
	ALIC-L	NA	NA	(T5)-0.891353	0.0422815
	ST-R	NA	NA	(T6)0.859125	0.06211
	AA-WM-R	NA	NA	(T3)0.90225	0.026141
FA	CP-R	NA	NA	(T3)0.9260468	0.0238721
	CP-L	NA	NA	(T5)0.900711	0.0369919

NA means without significant correlation. GCC, Genu of Corpus Callosum; ALIC-R, Anterior Limb of the Internal Capsule - Right; PLIC-L, Posterior Limb of the Internal Capsule - Left; ALIC-L, Anterior Limb of the Internal Capsule - Left; ST-R, Stria Terminalus - Right; AA-WM-R, Adjacent Amygdala White Matter - Right; CP-R, Cerebral Peduncle - Right; CP-L, Cerebral Peduncle - Left. T3 means time point 3 (that is 5 weeks post inoculation); T5 means time point 5 (that is 24 weeks post inoculation); T6 means time point 6 (that is 36 weeks post inoculation).

**Table 2:** The correlations between DTI metrics and viral load with regular cART treatment.

	Target region	plasma viral load		CSF viral load	
		r	p	r	p
MD	PLIC-L	(T4)-0.893685	0.040942	NA	NA
	PLIC-R	(T4)-0.87555	0.0517050	NA	NA
	GCC	(T4)-0.930233	0.0218882	NA	NA
	BCC	(T4)-0.899352	0.03774611	NA	NA
	SS-R	(T6)-0.974256953	0.004939	NA	NA
	CP-R	NA	NA	(T5)0.9898	0.001219
FA	ALIC-R	(T6)0.92219	0.02574772	(T5)-0.9029	0.03574
	ALIC-L	NA	NA	(T5)-0.9402	0.0173
	ST-R	(T4)0.921946	0.0258683	NA	NA
	GCC	NA	NA	(T5)-0.9227	0.02549
	BCC	NA	NA	(T5)-0.9389	0.01793
	PLIC-L	NA	NA	(T5)-0.9580	0.01024
	PLIC-R	NA	NA	(T5)-0.9803	0.01731
	FX	NA	NA	(T5)-0.9271	0.02336
	CP-R	NA	NA	(T5)-0.9737	0.005086
	CP-L	NA	NA	(T5)-0.9888	0.001416
	SS-R	NA	NA	(T5)-0.9909	0.001041

PLIC-R, Posterior Limb of the Internal Capsule – Right; PLIC-L, Posterior Limb of the Internal Capsule – Left; GCC, Genu of Corpus Callosum; BCC, Body of Corpus Callosum; SS-R, Sagittal Striatum - Right; ALIC-R, Anterior Limb of the Internal Capsule – Right; ALIC-L, Anterior Limb of the Internal Capsule – Right; ST-R, Stria Terminalus – Right; FX, Fonix; CP-R, Cerebral Peduncle - Right; CP-L, Cerebral Peduncle - Left. T4 means time point 4 (that is 12 weeks post inoculation); T5 means time point 5 (that is 24 weeks post inoculation).

### 3.5 Correlation between altered DTI characteristics without treatment and clinical metrics

We also estimated the correlations between clinical metrics such as CD4 T cell count and CD4/CD8 ratio and altered DTI metrics with high significance. We found that decreased FA was associated with decreased numbers of CD4 T cells in PLIC-L, PLIC-R, and CP-R in monkeys without treatment. Increased MD was associated with low CD4 T cell counts in PLIC-R and PLIC-L, except for in STG-WM-L, where there was a positive relationship. For the CD4/CD8 ratio, an increase in MD was associated with a decrease in CD4/CD8 in PLIC-R, FX, and ST-R (**Table 3**). In addition, MD was positively correlated to plasma viral load in GCC ALIC-R, EC-L, and ST-R in SIV-infected macaques without treatment. CSF viral load showed a negative correlation with MD and in PLIC-R FX, and ST-R, and a positive correlation with FA (**Table 4**).

**Table 3:** The correlations between DTI metrics and clinical tests without treatment.

	Target region	CD4		CD4/CD8	
		r	p	r	p
MD	PLIC-R	(T1)-0.933691	0.0202919	NA	NA
	PLIC-L	(T1)-0.86411685	0.0588879	NA	NA
	STG-WM-L	(T6)0.9826859	0.0027277	NA	NA
	PLIC-R	NA	NA	(T1)-0.93372	0.02027519
	FX	NA	NA	(T1)-0.8782170	0.0500745
	ST-R	NA	NA	(T6)-0.865228	0.058177
FA	PLIC-L	(T1)0.946059	0.014916	NA	NA
	PLIC-R	(T1)0.9057661	0.034230	NA	NA
	FX	(T4)0.94168096	0.0167576	NA	NA

NA means without significant correlation. PLIC-R, Posterior Limb of the Internal Capsule – Right; PLIC-L, Posterior Limb of the Internal Capsule – Left; STG-WM-L, Superior Temporal Gyrus WM – Left; FX, Fornix; ST-R, Stria Terminalus – Right; CP-R, Cerebral Peduncle – Right. T1 means time point 1 (that is the baseline); T6 means time point 6 (that is 36 weeks post inoculation).

**Table 4:** The correlations between DTI metrics and viral load without treatment.

	Target region	plasma viral load		CSF viral load	
		r	p	r	p
MD	GCC	(T5)0.883347	0.046981	NA	NA
	BCC	NA	NA	(T6)-0.9327	0.02070
	ALIC-R	(T5)0.945656	0.0150828	(T6) -0.92378	0.02497
	EC-L	(T5)0.943643	0.015923	NA	NA
	ST-R	(T2)0.8792436	0.04945	NA	NA
	PLIC-R	NA	NA	(T5)-0.8961	0.03954
	ALIC-L	NA	NA	(T6)-0.96057	0.009344
	CP-R	NA	NA	(T6)-0.9431	0.01614
	EC-L	NA	NA	(T4)0.8908	0.04256
	SS-R	NA	NA	(T6)-0.9764	0.004314
FA	CP-L	NA	NA	(T6)0.9552	0.01128
	ALIC-L	NA	NA	(T6)0.98519	0.002158

GCC, Genu of Corpus Callosum; BCC , Body of Corpus Callosum; ALIC-R, Anterior Limb of the Internal Capsule–Right; ST-R, Stria Terminalus–Right; PLIC-R, Posterior Limb of the Internal Capsule–Right; ALIC-L, Anterior Limb of the Internal Capsule–Left; CP-R, Cerebral Peduncle - Right; CP-L, Cerebral Peduncle–Left; SS-R, Sagittal Striatum - Right; EC-L, External Capsule - Left. T2 means time point 2 (that is 1 week post inoculation); T5 means time point 5 (that is 24 weeks post inoculation); T6 means time point 6 (that is 36 weeks post inoculation).

## 4 Discussion

The current study investigated longitudinal structural changes in brain white matter in 10 SIV-mac239-infected monkeys with and without regular cART treatment. We found significant white matter structural changes in these monkeys that consisted of higher mean diffusion and lower fractional anisotropy, as determined by DTI. The results showed that these effects increased with time. Furthermore, we found that significant white matter disruption occurred as early as 4 weeks after inoculation and that damage could be mitigated or even reversed with regular cART. In addition, DTI metric changes correlated significantly with clinical measures such as CD4 T cell count, CD4/CD8, plasma viral load, and CSF viral load.

The etiology underlying white matter changes in HIV-infected individuals is still unclear. Possible pathophysiological mechanisms include synaptodendritic damage, inflammation, demyelination, and even microvascular abnormalities that correlate with concomitant cardiovascular risk factors, particularly hypertension. In the context of HIV, on the basis of DTI acquisition parameters, FA reflects the axonal structural integrity aspect, which reflects the cytoskeleton and membrane integrity (34) and the structural integrity of the ricytes (35). MD has been interpreted to be related to the degree of WM microstructural density (34, 36), such as the extracellular space between WM tracts (35), which may be affected to a lesser extent by neuroinflammation, including glia involvement and myelin loss. In this case, the contribution of myelin to MD changes is questionable (35) because massive demyelination is not a common feature of HIV-related brain injury, even in patients with severe HIV encephalopathy (37). Previous studies in HIV patients and healthy controls have shown subtle but widespread white matter alterations, particularly changes in fractional anisotropy and mean diffusion (9, 11, 19, 38–45), suggesting the disorganization of white matter micro- and macrostructures (46).

Few studies have, however, investigated the initial timing of the changes, groups of regions in which white matter is first destroyed, and what happens after early cART (as early as 40 days after inoculation), which may shed light on the underlying neuropathological mechanisms and illustrate the impact of timely cART on imaging findings, immune indicators, and the relationship between them. In our study, we longitudinally examined white matter changes in rhesus macaques with and without regular cART treatment from baseline and 10 days, 4 weeks, 12 weeks, 24 weeks, and 36 weeks after inoculation. In the present study, changes were first observed 4 weeks after inoculation in the amygdala, superior temporal gyrus (STG), posterior part of the internal capsule (left), fornix, bilateral brainstem, and bilateral anterior limb of the internal capsule (ALIC-L) at FA, and the posterior limb of the internal capsule-left (PLIC-L) and right sagittal striatum (SS -R) at MD, suggesting that these regions are more susceptible to SIV infection. Studies have shown that HIV enters the central nervous system very early after seroconversion *via* monocytes and perivascular macrophages. The virus then replicates and causes neuronal damage mainly through neuroinflammatory mechanisms triggered by infected microglial cells. Inflammation occurs selectively in dopamine-rich areas of the brain, including subcortical areas, particularly the dopamine-rich basal ganglia, leading to subcortical dementia (47–50). Our findings that FA alternates in the amygdala and sagittal striatum, which are part of the basal ganglia, are consistent with this work. The fornix is an important hippocampal outflow tract, a structure that is critical for normal memory function and has been reported to be abnormal in AD, schizophrenia, and multiple sclerosis. Microstructural alterations to the fornix contribute to early episodic memory impairment in non-demented individuals (51–54). The internal capsule is a region that is sensitive to HIV and AIDS in individuals with cognitive impairment (55). A previous study also reported that a higher HIV RNA density of infected macrophages was found in the subcortical white matter, including IC (56). In addition, many studies also reported white matter damage in the subcortical white matter, especially the inner capsule (11, 12, 40, 57). Regarding alterations to the cerebellar peduncle and the stratum sagittale, similar changes have been found in previous studies in adolescents (58).

Twelve weeks after inoculation, the PLIC-R, GCC, BCC, EC-L, and ST-R regions showed significant changes at FA, and PLIC-R was altered at MD. Early cerebral infiltration of HIV may occur before antiretroviral drugs are administered, exposing the surrounding white matter to neurotoxic effects before effective viral suppression occurs. Periventricular white matter, such as CC, has been suggested to be particularly vulnerable to viral attack because of its proximity to the CSF, which is an HIV reservoir that can transmit the virus within 8 days of infection (59–61). Studies have also shown that the highest density of HIV-infected macrophages is found in the subcortical white matter, including the CC (56). Consistent with these early effects on the brain, white matter depletion has been found within the first 100 days of HIV transmission (62). As an important communication pathway between hemispheres, the CC is responsible for the functional integration of complex cognitive, motor, and behavioral tasks. Therefore, observations of the CC may indicate the presence of an underlying neurocognitive disorder in HIV-infected individuals. The outer capsule serves as a pathway for psychomotor functions, and many previous studies have shown the outer capsule to be altered in HIV patients (63, 64).

After cART, we found that the initial changes disappeared in BCC and FX; there was no progression in AA-WM-R, PLIC-R, or PLIC-L, slow progression in STG-WM-L, GCC, CP-R, and CP-L, and no effect on PLICR, ALIC-R, SS-R, or ST-R. In contrast, in EC-L, we found that cART damaged the structure to some extent. White matter repair by viral suppression and cART could lead to a higher degree of axonal structural integrity than the normal effects of aging. This may be particularly possible in the inner capsule because this is a region that is susceptible to white matter damage in individuals with cognitive impairment and AIDS (55). The absence of aggressive HIV-associated WM damage in the CC after cART suggests that early treatment is neuroprotective to some extent (65). On the one hand, cART may contribute to synaptic damage *via* oxidative stress, as demonstrated *in vitro* and in animal models (66). On the other hand, early and continuous antiretroviral therapy may be neuroprotective and limit white matter damage (67, 68).

Viral load levels reflect the replication of HIV virus in the body and can be used to determine disease progression and the effectiveness of antiviral treatment. A negative relationship was found between DTI parameters and viral load in SIV-mac239 infected rhesus monkeys with and without cART treatment, which is relatively consistent with a prior study that showed that myeloid cells, particularly microglia, are likely to be major reservoirs in the brain, and that more microglia exist in white matter (69). Activation of both infected and uninfected macrophages and microglia in the brain could promote inflammation and edema and affect DTI metrics. Specifically, the greater amount of inflammatory cells and surrounding debris could restrict water diffusion and lead to alterations in both FA and MD (30, 31), suggesting that the increased diffusion may reflect increased glial activation or inflammation. However, this suggestion would be made more convincing by appropriate pathologic studies.

In the clinic, current CD4 T cell counts are used as markers to monitor current immune functions and track disease progression (70). CD4/CD8 ratio is a marker of persistent inflammation and immunosenescence caused by viral infections (71). In our study, we found a decreased number of CD4 T cell counts were associated with a decreased FA value and an increased MD value in patients without cART treatment, which was also seen in a prior study (72), indicating the close relationship between CD4 T cell counts and white matter alterations, although the underlying the mechanism is not completely understood. After early and regular cART treatment, there were no significant changes in CD4 T cell counts or DTI metrics, suggesting that absolute CD4 T cell counts may not accurately reflect the risks to HIV-infected individuals because immune dysfunction persists even when CD4 counts are back to normal (73). Furthermore, we found that increased MD was associated with decreased CD4/CD8 in many brain regions in macaques with and without regular cART treatment, which is consistent with prior studies in humans and macaques (29, 60). A low CD4/CD8 ratio is an immunologic risk phenotype associated with altered immune senescence, immune function, and chronic inflammation. After inoculation with SIV, a sharp increase in inflammatory cytokine production may indirectly lead to white matter damage, suggesting that CD4/CD8 in cART eara may be a better biomarker of disease progression, treatment response, morbidity, and mortality in virally suppressed individuals (74).

### 4.1 Limitations and further considerations

First, cognitive performance was not recorded in our study because it was very time consuming to train the monkeys on specific tasks. This prevented us from investigating the underlying mechanisms of neurocognitive dysfunction and correlations with DTI metrics and clinical tests. Second, we only examined early structural changes in the brain. The results of a longer follow-up period may be of importance, and we will continue follow-up and observation. In addition, the small sample size limited our ability to perform effective statistical analyzes,and we also did not perform pathological anatomic studies. Therefore, in a future study, we will include a larger sample of macaques and examine their pathological anatomy.

## 5 Conclusions

Animals infected with SIV-mac239 may be ideal models for studying HIV-induced, HIV-associated brain changes. In our model, the first white matter changes appeared in a structure adjacent to the periventricular area as early as 4 weeks after inoculation. The changes progressed over time, and cART treatment may have had different effects on each region, including reversing, alleviating, and even progressive effects. These changes were closely related to the CD4/CD8 ratio and viral load even after cART. Further studies are urgently needed to elucidate the underlying mechanisms.

## Data availability statement

The original contributions presented in the study are included in the article/**Supplementary Material**. Further inquiries can be directed to the corresponding authors.

## Ethics statement

The animal study was reviewed and approved by the Institutional Animal Care and Use Committee (IACUC) at the Institute of Laboratory Animal Science, Chinese Academy of Medical Sciences (IACUC Approval No: LHJ18001).

## Author contributions

Conceptualization: JL. Data collection: DL, YQ, XA, SH, YG. Methodology: BN. Data analysis: JL, BN. Supervision: HL. Writing—original draft: JL, BN. Writing—review & editing: XH, HL. All authors contributed to the article and approved the submitted version.
