# Supplementary material for: Longitudinal white matter alterations in SIVmac239-infected rhesus monkeys with and without regular cART treatment
Source: Front Immunol. 2023 Jan 12;13:1067795. doi: 10.3389/fimmu.2022.1067795 (PMC9879061; doi:10.3389/fimmu.2022.1067795)
Supplement: Supplementary file 1 [file Table_1.docx]

| **GCC** | MK1(cART-) | MK2(cART-) | MK3(cART-) | MK6(cART-) | MK12(cART-) | MK5(cART+) | MK7(cART+) | MK9(cART+) | MK10(cART+) | MK11(cART+) |
| --- | --- | --- | --- | --- | --- | --- | --- | --- | --- | --- |
| T1 | 0.000531 | 0.000571 | 0.000631 | 0.000579 | 0.000629 | 0.000608 | 0.000588 | 0.000545 | 0.0006 | 0.000546 |
| T2 | 0.000528 | 0.000566 | 0.000583 | 0.000573 | 0.00063 | 0.000518 | 0.000572 | 0.000542 | 0.000597 | 0.000544 |
| T3 | 0.000512 | 0.000557 | 0.000584 | 0.000495 | 0.000593 | 0.00059 | 0.000545 | 0.000561 | 0.000565 | 0.000509 |
| T4 | 0.000536 | 0.000583 | 0.000608 | 0.000561 | 0.000617 | 0.000656 | 0.000609 | 0.000526 | 0.00055 | 0.000552 |
| T5 | 0.000543 | 0.000489 | 0.000623 | 0.00058 | 0.000652 | 0.000593 | 0.000566 | 0.000528 | 0.000587 | 0.000568 |
| T6 | 0.000578 | 0.000573 | 0.000608 | 0.000573 | 0.00063 | 0.000632 | 0.000585 | 0.00056 | 0.000605 | 0.000562 |
| **BCC** | MK1(cART-) | MK2(cART-) | MK3(cART-) | MK6(cART-) | MK12(cART-) | MK5(cART+) | MK7(cART+) | MK9(cART+) | MK10(cART+) | MK11(cART+) |
| T1 | 0.447942 | 0.470800 | 0.372001 | 0.506729 | 0.447318 | 0.354469 | 0.461148 | 0.500213 | 0.468670 | 0.462460 |
| T2 | 0.477875 | 0.363100 | 0.350072 | 0.492955 | 0.415846 | 0.245773 | 0.428138 | 0.502673 | 0.457370 | 0.454587 |
| T3 | 0.438775 | 0.337702 | 0.348492 | 0.334584 | 0.331114 | 0.245448 | 0.415618 | 0.491340 | 0.415292 | 0.432535 |
| T4 | 0.449549 | 0.501347 | 0.382690 | 0.526126 | 0.455544 | 0.346783 | 0.466982 | 0.548013 | 0.472616 | 0.465079 |
| T5 | 0.461581 | 0.403560 | 0.354785 | 0.533356 | 0.451311 | 0.295908 | 0.447228 | 0.529359 | 0.473238 | 0.470658 |
| T6 | 0.479875 | 0.457109 | 0.391829 | 0.551714 | 0.408727 | 0.381626 | 0.456579 | 0.569761 | 0.513732 | 0.444927 |
| **CP-R** | MK1(cART-) | MK2(cART-) | MK3(cART-) | MK6(cART-) | MK12(cART-) | MK5(cART+) | MK7(cART+) | MK9(cART+) | MK10(cART+) | MK11(cART+) |
| T1 | 0.468604 | 0.526774 | 0.482363 | 0.575658 | 0.515469 | 0.432738 | 0.514699 | 0.524592 | 0.488959 | 0.565259 |
| T2 | 0.510758 | 0.371530 | 0.444509 | 0.499751 | 0.504240 | 0.269772 | 0.485419 | 0.476649 | 0.545186 | 0.571585 |
| T3 | 0.441906 | 0.288668 | 0.417929 | 0.282639 | 0.278504 | 0.289465 | 0.443054 | 0.463063 | 0.329689 | 0.392408 |
| T4 | 0.505354 | 0.560497 | 0.581952 | 0.575963 | 0.582297 | 0.494774 | 0.541191 | 0.616362 | 0.587560 | 0.557244 |
| T5 | 0.552150 | 0.454382 | 0.472783 | 0.540333 | 0.579411 | 0.355444 | 0.514249 | 0.563376 | 0.558799 | 0.560479 |
| T6 | 0.584898 | 0.525087 | 0.510436 | 0.549305 | 0.540112 | 0.561476 | 0.541460 | 0.569290 | 0.561109 | 0.502123 |
| **CP-L** | MK1(cART-) | MK2(cART-) | MK3(cART-) | MK6(cART-) | MK12(cART-) | MK5(cART+) | MK7(cART+) | MK9(cART+) | MK10(cART+) | MK11(cART+) |
| T1 | 0.441686 | 0.512904 | 0.482842 | 0.555342 | 0.495538 | 0.405398 | 0.511852 | 0.530898 | 0.499293 | 0.556711 |
| T2 | 0.507103 | 0.351488 | 0.423830 | 0.537889 | 0.473556 | 0.308006 | 0.485635 | 0.455499 | 0.559149 | 0.549927 |
| T3 | 0.384232 | 0.272430 | 0.401283 | 0.196081 | 0.233749 | 0.261617 | 0.404869 | 0.453090 | 0.284582 | 0.346054 |
| T4 | 0.499621 | 0.579492 | 0.533616 | 0.539064 | 0.565647 | 0.449213 | 0.558775 | 0.560652 | 0.498375 | 0.517074 |
| T5 | 0.538001 | 0.433801 | 0.485310 | 0.574456 | 0.577467 | 0.314518 | 0.516072 | 0.547521 | 0.517245 | 0.512455 |
| T6 | 0.524620 | 0.500474 | 0.511431 | 0.570911 | 0.502478 | 0.538225 | 0.521098 | 0.543519 | 0.531016 | 0.522809 |
| **ALIC-R** | MK1(cART-) | MK2(cART-) | MK3(cART-) | MK6(cART-) | MK12(cART-) | MK5(cART+) | MK7(cART+) | MK9(cART+) | MK10(cART+) | MK11(cART+) |
| T1 | 0.441686 | 0.512904 | 0.482842 | 0.555342 | 0.495538 | 0.405398 | 0.511852 | 0.530898 | 0.499293 | 0.556711 |
| T2 | 0.507103 | 0.351488 | 0.423830 | 0.537889 | 0.473556 | 0.308006 | 0.485635 | 0.455499 | 0.559149 | 0.549927 |
| T3 | 0.384232 | 0.272430 | 0.401283 | 0.196081 | 0.233749 | 0.261617 | 0.404869 | 0.453090 | 0.284582 | 0.346054 |
| T4 | 0.499621 | 0.579492 | 0.533616 | 0.539064 | 0.565647 | 0.449213 | 0.558775 | 0.560652 | 0.498375 | 0.517074 |
| T5 | 0.538001 | 0.433801 | 0.485310 | 0.574456 | 0.577467 | 0.314518 | 0.516072 | 0.547521 | 0.517245 | 0.512455 |
| T6 | 0.524620 | 0.500474 | 0.511431 | 0.570911 | 0.502478 | 0.538225 | 0.521098 | 0.543519 | 0.531016 | 0.522809 |
| **ALIC-L** | MK1(cART-) | MK2(cART-) | MK3(cART-) | MK6(cART-) | MK12(cART-) | MK5(cART+) | MK7(cART+) | MK9(cART+) | MK10(cART+) | MK11(cART+) |
| T1 | 0.362915 | 0.377459 | 0.351306 | 0.376950 | 0.390092 | 0.287015 | 0.378301 | 0.384187 | 0.367296 | 0.404048 |
| T2 | 0.400361 | 0.277810 | 0.320664 | 0.373672 | 0.362567 | 0.224612 | 0.353413 | 0.377730 | 0.394997 | 0.412027 |
| T3 | 0.329159 | 0.271249 | 0.312790 | 0.221027 | 0.251904 | 0.239700 | 0.329964 | 0.370871 | 0.265833 | 0.319064 |
| T4 | 0.374324 | 0.414233 | 0.381515 | 0.381788 | 0.391704 | 0.289437 | 0.405931 | 0.417709 | 0.366896 | 0.402431 |
| T5 | 0.390776 | 0.313015 | 0.350023 | 0.415020 | 0.400100 | 0.242086 | 0.364364 | 0.436674 | 0.397213 | 0.404395 |
| T6 | 0.391861 | 0.361980 | 0.378025 | 0.421277 | 0.366245 | 0.347043 | 0.388392 | 0.427094 | 0.386782 | 0.384215 |
| **PLIC-R** | MK1(cART-) | MK2(cART-) | MK3(cART-) | MK6(cART-) | MK12(cART-) | MK5(cART+) | MK7(cART+) | MK9(cART+) | MK10(cART+) | MK11(cART+) |
| T1 | 0.478885 | 0.503323 | 0.451525 | 0.545121 | 0.508073 | 0.447043 | 0.489771 | 0.563929 | 0.486298 | 0.516101 |
| T2 | 0.515009 | 0.290532 | 0.445885 | 0.499701 | 0.514395 | 0.217584 | 0.498281 | 0.514733 | 0.545675 | 0.532554 |
| T3 | 0.438437 | 0.226874 | 0.438326 | 0.283781 | 0.261310 | 0.198633 | 0.445907 | 0.517667 | 0.346385 | 0.406426 |
| T4 | 0.497366 | 0.529960 | 0.532054 | 0.565113 | 0.534512 | 0.472314 | 0.530492 | 0.613740 | 0.529766 | 0.532649 |
| T5 | 0.506158 | 0.417572 | 0.475015 | 0.547960 | 0.539257 | 0.383065 | 0.516804 | 0.569523 | 0.511851 | 0.517198 |
| T6 | 0.553344 | 0.473782 | 0.494163 | 0.551212 | 0.523330 | 0.542986 | 0.530292 | 0.562036 | 0.504946 | 0.486619 |
| **PLIC-L** | MK1(cART-) | MK2(cART-) | MK3(cART-) | MK6(cART-) | MK12(cART-) | MK5(cART+) | MK7(cART+) | MK9(cART+) | MK10(cART+) | MK11(cART+) |
| T1 | 0.494733 | 0.489240 | 0.465951 | 0.571515 | 0.526248 | 0.391761 | 0.503044 | 0.504006 | 0.502806 | 0.534618 |
| T2 | 0.523196 | 0.265428 | 0.417307 | 0.531900 | 0.495605 | 0.200720 | 0.501198 | 0.469001 | 0.546988 | 0.541174 |
| T3 | 0.407408 | 0.226614 | 0.417303 | 0.243996 | 0.295050 | 0.199484 | 0.437211 | 0.485858 | 0.330137 | 0.404126 |
| T4 | 0.510631 | 0.541188 | 0.512915 | 0.549395 | 0.543363 | 0.423117 | 0.536525 | 0.572331 | 0.528386 | 0.532781 |
| T5 | 0.551802 | 0.333692 | 0.479533 | 0.562291 | 0.538160 | 0.319455 | 0.506730 | 0.572545 | 0.502636 | 0.525788 |
| T6 | 0.530880 | 0.440471 | 0.489780 | 0.566366 | 0.521762 | 0.518582 | 0.524478 | 0.526570 | 0.532957 | 0.532546 |
| **SS-R** | MK1(cART-) | MK2(cART-) | MK3(cART-) | MK6(cART-) | MK12(cART-) | MK5(cART+) | MK7(cART+) | MK9(cART+) | MK10(cART+) | MK11(cART+) |
| T1 | 0.494733 | 0.489240 | 0.465951 | 0.571515 | 0.526248 | 0.391761 | 0.503044 | 0.504006 | 0.502806 | 0.534618 |
| T2 | 0.523196 | 0.265428 | 0.417307 | 0.531900 | 0.495605 | 0.200720 | 0.501198 | 0.469001 | 0.546988 | 0.541174 |
| T3 | 0.407408 | 0.226614 | 0.417303 | 0.243996 | 0.295050 | 0.199484 | 0.437211 | 0.485858 | 0.330137 | 0.404126 |
| T4 | 0.510631 | 0.541188 | 0.512915 | 0.549395 | 0.543363 | 0.423117 | 0.536525 | 0.572331 | 0.528386 | 0.532781 |
| T5 | 0.551802 | 0.333692 | 0.479533 | 0.562291 | 0.538160 | 0.319455 | 0.506730 | 0.572545 | 0.502636 | 0.525788 |
| T6 | 0.530880 | 0.440471 | 0.489780 | 0.566366 | 0.521762 | 0.518582 | 0.524478 | 0.526570 | 0.532957 | 0.532546 |
| **EC-L** | MK1(cART-) | MK2(cART-) | MK3(cART-) | MK6(cART-) | MK12(cART-) | MK5(cART+) | MK7(cART+) | MK9(cART+) | MK10(cART+) | MK11(cART+) |
| T1 | 0.286470 | 0.325434 | 0.233976 | 0.330224 | 0.295073 | 0.251706 | 0.277615 | 0.259390 | 0.246724 | 0.311542 |
| T2 | 0.327290 | 0.234288 | 0.221473 | 0.290700 | 0.309760 | 0.214438 | 0.275906 | 0.268652 | 0.267752 | 0.315487 |
| T3 | 0.291590 | 0.219427 | 0.228903 | 0.190275 | 0.260881 | 0.175425 | 0.241616 | 0.257743 | 0.194509 | 0.258272 |
| T4 | 0.304801 | 0.358650 | 0.305322 | 0.298985 | 0.303549 | 0.250002 | 0.309551 | 0.293026 | 0.263787 | 0.322313 |
| T5 | 0.313116 | 0.258653 | 0.225768 | 0.295586 | 0.321452 | 0.230515 | 0.277524 | 0.295248 | 0.271195 | 0.313226 |
| T6 | 0.310146 | 0.305274 | 0.222899 | 0.309491 | 0.299263 | 0.291548 | 0.294896 | 0.300145 | 0.279812 | 0.299851 |

| **CP-L** | MK1(cART-) | MK2(cART-) | MK3(cART-) | MK6(cART-) | MK12(cART-) | MK5(cART+) | MK7(cART+) | MK9(cART+) | MK10(cART+) | MK11(cART+) |
| --- | --- | --- | --- | --- | --- | --- | --- | --- | --- | --- |
| T1 | 0.000565 | 0.000552 | 0.000537 | 0.000513 | 0.000565 | 0.000593 | 0.000571 | 0.000531 | 0.000556 | 0.000512 |
| T2 | 0.000535 | 0.000590 | 0.000567 | 0.000523 | 0.000583 | 0.000459 | 0.000540 | 0.000568 | 0.000516 | 0.000516 |
| T3 | 0.000594 | 0.000599 | 0.000581 | 0.000638 | 0.000679 | 0.000547 | 0.000570 | 0.000580 | 0.000590 | 0.000597 |
| T4 | 0.000546 | 0.000501 | 0.000532 | 0.000542 | 0.000538 | 0.000577 | 0.000553 | 0.000536 | 0.000532 | 0.000542 |
| T5 | 0.000524 | 0.000517 | 0.000539 | 0.000501 | 0.000544 | 0.000579 | 0.000514 | 0.000503 | 0.000546 | 0.000528 |
| T6 | 0.000542 | 0.000553 | 0.000534 | 0.000538 | 0.000537 | 0.000543 | 0.000518 | 0.000532 | 0.000535 | 0.000523 |
| **PLIC-R** | MK1(cART-) | MK2(cART-) | MK3(cART-) | MK6(cART-) | MK12(cART-) | MK5(cART+) | MK7(cART+) | MK9(cART+) | MK10(cART+) | MK11(cART+) |
| T1 | 0.000525 | 0.000527 | 0.000522 | 0.000496 | 0.000511 | 0.000522 | 0.000506 | 0.000488 | 0.000505 | 0.000505 |
| T2 | 0.000521 | 0.000559 | 0.000522 | 0.000506 | 0.000520 | 0.000549 | 0.000491 | 0.000520 | 0.000500 | 0.000505 |
| T3 | 0.000533 | 0.000557 | 0.000524 | 0.000530 | 0.000553 | 0.000593 | 0.000512 | 0.000499 | 0.000524 | 0.000532 |
| T4 | 0.000523 | 0.000507 | 0.000494 | 0.000487 | 0.000502 | 0.000533 | 0.000504 | 0.000464 | 0.000501 | 0.000495 |
| T5 | 0.000499 | 0.000492 | 0.000490 | 0.000477 | 0.000507 | 0.000515 | 0.000472 | 0.000481 | 0.000503 | 0.000515 |
| T6 | 0.000492 | 0.000520 | 0.000510 | 0.000487 | 0.000486 | 0.000502 | 0.000491 | 0.000507 | 0.000508 | 0.000505 |
| **PLIC-L** | MK1(cART-) | MK2(cART-) | MK3(cART-) | MK6(cART-) | MK12(cART-) | MK5(cART+) | MK7(cART+) | MK9(cART+) | MK10(cART+) | MK11(cART+) |
| T1 | 0.000533 | 0.000521 | 0.000522 | 0.000490 | 0.000513 | 0.000540 | 0.000514 | 0.000505 | 0.000507 | 0.000505 |
| T2 | 0.000510 | 0.000569 | 0.000537 | 0.000514 | 0.000549 | 0.000545 | 0.000492 | 0.000528 | 0.000500 | 0.000503 |
| T3 | 0.000556 | 0.000555 | 0.000539 | 0.000544 | 0.000570 | 0.000598 | 0.000532 | 0.000517 | 0.000531 | 0.000555 |
| T4 | 0.000527 | 0.000504 | 0.000499 | 0.000499 | 0.000520 | 0.000545 | 0.000513 | 0.000476 | 0.000503 | 0.000496 |
| T5 | 0.000498 | 0.000518 | 0.000515 | 0.000489 | 0.000524 | 0.000529 | 0.000479 | 0.000474 | 0.000516 | 0.000509 |
| T6 | 0.000511 | 0.000546 | 0.000508 | 0.000498 | 0.000508 | 0.000505 | 0.000488 | 0.000508 | 0.000520 | 0.000499 |
| **SS-R** | MK1(cART-) | MK2(cART-) | MK3(cART-) | MK6(cART-) | MK12(cART-) | MK5(cART+) | MK7(cART+) | MK9(cART+) | MK10(cART+) | MK11(cART+) |
| T1 | 0.000555 | 0.000585 | 0.000588 | 0.000574 | 0.000593 | 0.000576 | 0.000554 | 0.000578 | 0.000575 | 0.000567 |
| T2 | 0.000549 | 0.000606 | 0.000590 | 0.000580 | 0.000614 | 0.000604 | 0.000552 | 0.000596 | 0.000577 | 0.000569 |
| T3 | 0.000580 | 0.000602 | 0.000593 | 0.000603 | 0.000637 | 0.000648 | 0.000560 | 0.000615 | 0.000602 | 0.000605 |
| T4 | 0.000570 | 0.000579 | 0.000562 | 0.000570 | 0.000607 | 0.000640 | 0.000554 | 0.000556 | 0.000561 | 0.000574 |
| T5 | 0.000549 | 0.000570 | 0.000559 | 0.000546 | 0.000588 | 0.000607 | 0.000538 | 0.000553 | 0.000553 | 0.000581 |
| T6 | 0.000568 | 0.000587 | 0.000577 | 0.000551 | 0.000599 | 0.000578 | 0.000577 | 0.000561 | 0.000574 | 0.000579 |
| **STG-L** | MK1(cART-) | MK2(cART-) | MK3(cART-) | MK6(cART-) | MK12(cART-) | MK5(cART+) | MK7(cART+) | MK9(cART+) | MK10(cART+) | MK11(cART+) |
| T1 | 0.000542 | 0.000556 | 0.000573 | 0.000563 | 0.000607 | 0.000591 | 0.000562 | 0.000564 | 0.000577 | 0.000592 |
| T2 | 0.000558 | 0.000667 | 0.000599 | 0.000574 | 0.000634 | 0.000621 | 0.000556 | 0.000580 | 0.000579 | 0.000585 |
| T3 | 0.000603 | 0.000661 | 0.000577 | 0.000653 | 0.000665 | 0.000685 | 0.000581 | 0.000607 | 0.000597 | 0.000645 |
| T4 | 0.000536 | 0.000533 | 0.000563 | 0.000590 | 0.000595 | 0.000582 | 0.000565 | 0.000525 | 0.000559 | 0.000580 |
| T5 | 0.000539 | 0.000589 | 0.000578 | 0.000550 | 0.000626 | 0.000634 | 0.000534 | 0.000529 | 0.000567 | 0.000582 |
| T6 | 0.000533 | 0.000576 | 0.000557 | 0.000558 | 0.000615 | 0.000563 | 0.000566 | 0.000562 | 0.000581 | 0.000583 |
